# Mitochondrial complex I and III gene mRNA levels in schizophrenia, and their relationship with clinical features

**DOI:** 10.1186/s40303-014-0006-9

**Published:** 2014-12-10

**Authors:** Süleyman Akarsu, Deniz Torun, Abdullah Bolu, Murat Erdem, Salih Kozan, Mehmet Ak, Hatice Akar, Özcan Uzun

**Affiliations:** Department of Psychiatry, Aksaz Military Hospital, Marmaris, Muğla Turkey; Department of Medical Genetics, Gülhane Military Medical Faculty, Ankara, Turkey; Aircrew’s Health Research and Training Center, Eskişehir, Turkey; Department of Psychiatry, Gülhane Military Medical Faculty, Ankara, Turkey; Department of Psychiatry, Meram Faculty of Medicine, Necmettin Erbakan University, Konya, Turkey

**Keywords:** Schizophrenia, Mitochondrial dysfunction, Electron transport chain, Psychotic symptomatology, mRNA levels

## Abstract

**Background:**

The etiology of schizophrenia is not precisely known; however, mitochondrial function and cerebral energy metabolism abnormalities were determined to be possible factors associated with the etiology of schizophrenia. Impaired mitochondrial function negatively affects neuronal plasticity, and can cause cognitive deficits and behavioral abnormalities observed during the clinical course of schizophrenia. The present study aimed to investigate the relationship between the clinical features of schizophrenia, and mitochondrial complex activation, based on measurement of mRNA levels in the *NDUFV1*, *NDUFV2*, *NDUFS1*, and *UQCR10* genes involved in the peripheral mitochondrial complex.

**Methods:**

The study included 138 schizophrenia patients and 42 healthy controls. The schizophrenia group was divided into a chronic schizophrenia subgroup (n = 84) and a first-episode schizophrenia subgroup (n = 54). The symptoms profile and severity of disorder were evaluated using the Scale for the Assessment of Negative Symptoms (SANS), Scale for the Assessment of Positive Symptoms (SAPS), and Brief Psychiatric Rating Scale (BPRS).

**Results:**

The level of mRNA expression of *NDUFV1*, *NDUFV2*, and *NDUFS1* was significantly higher in the schizophrenia group than in the control group. The mRNA level of *NDUFV2* was positively correlated with BPRS and SAPS scores in the first-episode schizophrenia subgroup.

**Conclusion:**

The findings showed that there was a positive correlation between gene mRNA levels and psychotic symptomatology, especially positive symptoms. Our results suggest that mRNA levels of the *NDUFV1*, *NUDFV2*, and *NDUFS1* genes of complex I of the mitochondrial electron transport chain might become a possible peripheral marker for the diagnosis of schizophrenia.

## Background

Schizophrenia is a chronic psychiatric disorder and its etiology is not precisely known. A biomarker obtainable from peripheral tissues of schizophrenia patients would help to confirm the diagnosis and monitor its treatment. Mitochondrial function and cerebral energy metabolism abnormalities have been identified in patients with schizophrenia [1-4]. Impaired mitochondrial function negatively affects neuronal plasticity, and causes cognitive deficits and behavioral abnormalities that can be observed during the clinical course of schizophrenia [5].

Some studies that examined brain tissue in schizophrenia patients reported an increase in activation of the mitochondrial complex, as compared to controls [6,7], whereas others reported a decrease [8,9]. Location and function of the brain tissue examined were important in the formation of these results. Changes in mitochondrial complex activation in brain tissues occur based on positive or negative symptoms of schizophrenia. For example, activation of mitochondrial complex I subunits in prefrontal cortex neurons was low in the presence of hypofrontality characterized by negative symptoms of schizophrenia [9]. Variation in activation of mitochondrial complex I in different brain regions in schizophrenia patients is indicative of dysfunction of the pathways in the brain in schizophrenia [9].

The mitochondrial electron transport chain (mtETC) has been studied in an effort to find a biomarker for schizophrenia. Ben Shachar et al. [10] observed an increase in enzymatic activity of complex I in peripheral tissues in patients with schizophrenia, as compared to healthy controls. Dror et al. [5] reported that the observed increase in activation of mitochondrial complex I in patients with schizophrenia was associated with psychotic symptoms. Activation of mtETC complex I has been identified as a potential peripheral biomarker for schizophrenia [5,10].

It is important to understand the relationship between mitochondrial dysfunction and schizophrenia. A biomarker related to mitochondrial dysfunction obtainable from peripheral tissues in schizophrenia patients could help confirm the diagnosis; therefore, the present study aimed to investigate the relationship between clinical features and mRNA levels in certain genes located in the mtETC in schizophrenia patients.

## Methods

The study included 158 male schizophrenia patients aged 20–30 years with psychotic symptoms that were followed-up at the psychiatric clinic of a university hospital. Among the patients, 84 had been followed-up for schizophrenia for ≥1 year and were considered as chronic schizophrenia, and 74 had a negative psychiatric history, but exhibited schizophrenia-like psychotic symptoms for the first time and were followed-up for 6 months. After the sixth month of follow-up 54 of those 74 patients met Diagnostic and Statistical Manual of Mental Disorders (DSM)-IV [11] criteria for schizophrenia and were considered first-episode schizophrenia; the 20 patients that did not meet the diagnostic criteria for schizophrenia according to DSM-IV criteria were excluded from the study. The control group included 42 healthy male volunteers matched for age, level of education, and socioeconomic status. Exclusion criteria were any organic disease, history of psychoactive substance use, and use of any drug during the previous 3 months.

The study protocol was approved by the Gülhane Military Medical Academy Ethics Committee. All of the participants and their relatives were informed about the study and provided written informed consent. Blood samples were collected from the 158 patients and 42 healthy male volunteers. All the patients underwent a diagnostic interview using the Structured Clinical Interview for DSM-IV Axis I Disorders (SCID-I) [12]. The symptoms profile and severity of disease were evaluated using the Scale for the Assessment of Negative Symptoms (SANS), Scale for the Assessment of Positive Symptoms (SAPS), and Brief Psychiatric Rating Scale (BPRS). The SAPS scale consists of 30 items. It includes hallucinations, delusions, bizarre behavior, inappropriate affect and positive formal thought disorders [13]. The SANS scale comprises 25 items designed to assess five categories: blunted affect, alogia, apathy, anhedonia, and attention [14]. Both scales have a scoring range from 0 to 5. The BPRS assesses severity of 18 symptom constructs that are rated from 0 to 6. It shows baseline- prognosis of disorder and therapeutic responses [15]. Two trained senior psychiatrists performed the ratings and a consensus rating was obtained. Each of the three scales was performed prior to initiation of drug treatment to both groups of patients with schizophrenia. Blood samples were collected after the implementation of the scales on the same day. Messenger ribonucleic acid (mRNA) levels of genes from peripheral blood samples obtained from the participitants were investigated in the genetic laboratory.

### *NDUFV1*, *NDUFV2*, *NDUFS1*, and *UQCR10* Transcript Expression

Total RNA was extracted from peripheral blood samples according to standard protocols using an RNA Purification Kit (Norgen Biotek Corp., Canada). cDNA was synthesized from each RNA using a Transcriptor First Strand cDNA Synthesis Kit (Roche, Germany) using random hexamers, according to the manufacturer’s instructions. Quantitative PCR was performed using TaqMan probe sets (Real Time Ready Catalog Assay, Roche, Germany) specifically designed for *NDUFV1* (Assay ID: 142610), *NDUFV2* (Assay ID: 125807), *NDUFS1* (Assay ID: 128405), and *UQCR10* (Assay ID: 132402). The *β-actin* gene was used for normalization as an endogenous reference gene (Assay ID: 101125). Relative quantification was performed using Light Cycler Nano Software v.1.0. PCR cycling conditions were 95°C for 10 min, followed by 45 cycles at 95°C for 10 s, 60°C for 30 s, and 72°C for 30 s. Each sample was run in triplicate.

### Statistical analysis

Descriptive data are expressed as mean ± SD (range). Compliance with normal distribution for continuous variables was assessed via the Kolmogorov-Smirnov test. The Kruskal-Wallis test was used for comparison of multiple groups and the Mann–Whitney U test with Bonferroni correction was used as an advanced binary (post hoc) test. Comparison of the 2 groups was evaluated using the Mann–Whitney U test. Pearson’s correlation analysis was conducted to determine the relationship between the 2 independent variables. The level of statistical significance was set at P < 0.05.

## Results

### Clinical characteristics

Mean age at the time of assesment in the patient group was 22.3 ± 1.9 years (first-episode schizophrenia patients: 21.6 ± 1.5 years; chronic schizophrenia patients: 22.6 ± 3.9), versus 22.1 ± 1.9 years in the control group (P = 0.5). There was a significant difference in duration of disease (P < 0.01), age at disease onset (P < 0.01), and duration of hospitalization (P < 0.01) between the first-episode schizophrenia patients (n = 54) and the chronic schizophrenia patients (n = 84) (Table 1).

**Table 1:** **Clinical characteristics of schizophrenia cases**

**Variables**	**Groups**		**Statistical analysis**
	**First-episode schizophrenia (n = 54)**	**Chronic schizophrenia (n = 84)**	
Duration of disease; Mean ± s.d (month)	0.2 ± 0.1	32.2 ± 17.6	Z = −8.8; p < 0.01
Age of onset of the disease; Mean ± s.d (year)	21.6 ± 1.5	20.2 ± 1.7	Z = −3.8; p < 0.01
Duration of hospitalization; Mean ± s.d (day)	18.3 ± 3.5	14.4 ± 2.8	Z = −5.1; p < 0.01
SANS; Mean ± s.d	46.9 ± 11.9	48.2 ± 12.4	Z = −0.6; p = 0.55
SAPS; Mean ± s.d	58.7 ± 17.6	39.2 ± 21.9	Z = −4.1; p < 0.01
BPRS; Mean ± s.d	40.7 ± 9.4	31.0 ± 9.7	Z = −4.5; p < 0.01

Mean ± s.d : Mean ± standart deviation Z: Mann–Whitney U test.

No statistically significant difference was detected between first-episode schizophrenia and chronic schizophrenia in terms of SANS score (p = 0.55). SAPS (p < 0.01) and BPRS (p < 0.01) scores were higher in first-episode schizophrenia cases. The age of onset of the disease was higher in first-episode schizophrenia (p < 0.01). Duration of hospitalization was longer in patients with first-episode schizophrenia cases than chronic schizophrenia cases (p < 0.01).

### mRNA levels in mitochondrial complex I genes (*NDUFV1*, *NDUFV2*, and *NDUFS1*) and the complex III gene (*UQCR10*)

There was a significant difference in complex I gene mRNA levels between the patients and controls, as follows: *NDUFV1* (P = 0.01), *NDUFV2* (P < 0.01), and *NDUFS1* (P = 0.01), whereas the *UQCR10* gene (complex III) mRNA level did not differ between the groups (P = 0.7). There was not a significant difference in mRNA levels of *NDUFV1* (P = 0.09), *NDUFV2* (P = 0.28), *NDUFS1* (P = 0.32), or UQCR10 (P = 0.82) between the first-episode schizophrenia patients and chronic schizophrenia patients. There was a significant difference in the mRNA level of *NDUFV1* (P = 0.003), *NDUFV2* (P < 0.01), and *NDUFS1* (P = 0.003) between the first-episode schizophrenia patients and control subjects. There was also a significant difference in the mRNA level of *NDUFV2* (P < 0.01) between the chronic schizophrenia patients and controls (Table 2).

**Table 2:** **Gene mRNA levels of the cases**

**Variables**	**First episode schizophrenia (n = 54)**	**Chronic schizophrenia (n = 84)**	**Control (n = 42)**	**First episode vs chronic p**	**First episode vs control p**	**Cronic** **vs control p**
NDUFV1; Mean ± s.d	1.6 ± 1.3	1.3 ± 1.0	1.0 ± 0.0	Z = −1.7; 0.09	Z = −3.0; 0.003*	Z = −1.4; 0.2
NDUFV2; Mean ± s.d	2.0 ± 1.6	1.8 ± 1.5	1.0 ± 0.0	Z = −1.1; 0.28	Z = −5.0; < 0.01*	Z = −4.2; < 0.01*
NDUFS1; Mean ± s.d	1.6 ± 0.9	1.5 ± 1.0	1.0 ± 0.0	Z = −1.0; 0.32	Z = −3.0; 0.003*	Z = −2.8; 0,07
UQCR10; Mean ± s.d	5.7 ± 13.0	5.6 ± 17.5	1.0 ± 0.0	Z = −0.2; 0.82	Z = −6.7; 0.5	Z = −1.4; 0.2

Mean ± s.d: Mean ± standart deviation *p < 0.05 Z: Mann–Whitney U test.

mRNA levels of NDUFV1 were significantly higher in first-episode schizophrenia cases than control subjects (p = 0.003). mRNA levels of NDUFV2 were significantly higher in first-episode schizophrenia (p < 0.01) and chronic schizophrenia (p < 0.01) cases than control subjects. mRNA levels of NDUFS1 were significantly higher in first-episode schizophrenia cases than control subjects (p = 0.003).

### The relationship between mRNA levels of mitochondrial complex I genes (*NDUFV1*, *NDUFV2*, *NDUFS1*) and the complex III gene (*UQCR10*), and clinical characteristics

There was a positive significant correlation between the mRNA level of *NDUFV1* and age at onset of the disease (r = 0.21; P = 0.04), and a positive correlation between the mRNA level of *NDUFV2*, and BPRS score (r = 0.31, P = 0.03) and SAPS score (r = 0.2, P = 0.04) in the first-episode schizophrenia patients. The schizophrenia patients were also divided into subtype subgroups according to the predominance of psychotic symptoms: paranoid (n = 32), catatonic (n = 17), disorganized (n = 39), undifferentiated (n = 26), and residual (n = 24). There was not a difference in gene mRNA levels between the schizophrenia subtype subgroups (Table 3). As compared to the controls, mRNA levels of complex I genes (*NDUFV1*, *NDUFV2*, and *NDUFS1*) were significantly higher in the catatonic, disorganized, and undifferentiated patients (P < 0.05) (Table 3).

**Table 3:** **mRNA levels of the genes in schizophrenia subtypes**

**Variables**	**Paranoid (n = 32)**	**p** ^**a**^	**Catatonic (n = 17)**	**p** ^**a**^	**Disorganized (n = 39)**	**p** ^**a**^	**Undifferentiated (n = 26)**	**p** ^**a**^	**Residual (n = 24)**	**p** ^**a**^	**Statistical analysis**
NDUFV1; Mean ± s.d	1.4 ± 1.3	0.5	1.6 ± 0.7	0.01	1.7 ± 1.6	0.02	1.5 ± 1.2	0.02	1.3 ± 0.6	0.08	X ^2^ = 3.50; df = 2; p = 0.5
NDUFV2; Mean ± s.d	1.6 ± 1.2	0.2	2.1 ± 0.9	< 0.01	2.4 ± 1.9	< 0.01	1.7 ± 1.1	0.02	1.6 ± 1.8	0.1	X ^2^ = 5.48; df = 2; p = 0.2
NDUFS1; Mean ± s.d	1.3 ± 0.8	0.4	1.9 ± 1.0	0.01	1.7 ± 0.8	0.01	1.6 ± 0.7	< 0.01	1.5 ± 1.2	0,.2	X ^2^ = 4.10; df = 2; p = 0.4
UQCR10; Mean ± s.d	5.6 ± 13.5	0.6	12.9 ± 21.5	0.1	2.6 ± 4.2	0.7	1.8 ± 2.2	0.7	9.7 ± 24.8	0.2	X ^2^ = 2.5; df = 2; p = 0.6

Mean ± s.d: Mean ± standart deviation *X* ^*2*^: Kruskal Wallis test p^a^ : Compared with control group.

There was no significant difference in mRNA levels of schizophrenia subtypes. mRNA levels of complex I genes (NDUFV1, NDUFV2, NDUFS1) in catatonic, disorganized and undifferentiated types were significantly higher than control group.

## Discussion

As the etiology of schizophrenia remains unclear and its diagnosis is based on clinical criteria, there is a need for peripheral biological markers for schizophrenia. The present study’s findings show that there is a relationship between the clinical features of schizophrenia and the level of expressions of mitochondrial complex I genes.

### The mRNA level of mitochondrial complex genes

Activation of the mitochondrial complex was higher in schizophrenia patients than in controls in the majority of studies based on peripheral tissues. Such findings were interpreted to mean that an increase in activation of the mitochondrial complex could be considered a peripheral biomarker for schizophrenia [5,10,16,17]. The present study’s finding that increased mRNA levels of the 75-, 51-, and 24-kDa subunits (the largest subunits of mitochondrial complex I) in the patients with schizophrenia supported the hypothesis that an increase in activation of mitochondrial complex I might be a possible peripheral biomarker for schizophrenia. As there was not a difference in the mRNA level of the *UQCR10* gene (complex III) between the present study’s schizophrenic patients and controls, and the range of *UQCR10* mRNA levels was excessive, we do not consider activation of mitochondrial complex III an appropriate biomarker for schizophrenia.

In the present study the mRNA level of the 4 genes that were assessed in first-episode schizophrenia patients was higher than that in chronic schizophrenia patients, but the difference was not significant. The mRNA level of *NDUFV1*, *NDUFV2*, and *NDUFS1* in the first-episode schizophrenia patients was higher than that in the controls, and the mRNA level of *NDUFV2* was higher in the chronic schizophrenia patients than in the controls. SAPS and BPRS scores in the first-episode schizophrenia patients were higher than those in the chronic schizophrenia patients. These data suggest that there is a positive correlation between the level of gene mRNA expression and the clinical severity of schizophrenia. The present findings are in agreement with those of Dror et al. [5], who reported an increase in activation of complex I in patients with acute schizophrenia and a decrease in patients with residual schizophrenia. Dror et al.’s findings support the hypothesis that a decrease in activation of the mitochondrial complex is associated with clinical improvement in schizophrenia.

Furthermore, the mRNA level of *NDUFV2* was positively correlated with BPRS and SAPS scores in the present study’s first-episode schizophrenia patients. The positive correlation observed between the mRNA level of *NDUFV2* and SAPS score could be considered evidence that positive psychotic symptoms play a role in increasing the mRNA level, which is consistent with Dror et al.’s findings [5].

### The relationship between mRNA levels of the mitochondrial complexes and clinical characteristics

There was a moderate positive correlation between the mRNA level of *NDUFV1* and age at disease onset. In the present study patients of a particular age range were selected based on the potential effect of age on gene mRNA levels. Age at onset of disease in the present study’s schizophrenia patients was 17–26 years, and it was considered inappropriate to assess the relationship between gene mRNA levels and age at disease onset in this group; to properly evaluate this relationship comparison of gene mRNA levels in schizophrenia patients with early-onset and late-onset of symptoms would be more appropriate.

In the present study, a relationship between gene mRNA levels and duration of illness was not observed. As mRNA levels were similar in the first-episode and chronic schizophrenia patients, this finding is consistent with the comparison of first-episode and chronic schizophrenia patients with regard to gene mRNA levels. There were not any differences in the present study in gene mRNA levels of the genes according to the schizophrenia subtypes; this lack of difference supports the notion that the findings might reflect psychopathology of schizophrenia. Nonetheless, there were significant differences in mRNA levels of complex I genes between the controls, and the catatonic, disorganized, and undifferentiated subtype subgroups, whereas there was not a difference between the controls, and the paranoid or residual subtype subgroups. To the best our knowledge the present study is the first to compare mitochondria complex gene mRNA levels according to schizophrenia clinical subtypes. In paranoid and residual subtypes, behavioral pathologies, such as disorganized or catatonic expected to be less than the other types [11]. The present finding that gene mRNA levels were higher in the subtypes with behavioral pathology seen more common could be interpreted as there was a relationship between behavioral pathology and mRNA levels in schizophrenia, which is supported by earlier reports that changes in activation of the mitochondrial complex in neuronal cells leads to behavioral symptoms that cause abnormal neuronal transmission and synaptic plasticity [5,9,18-21].

### Limitations

Damage formation in mitochondrial structures increases with age [22-26]. The present study included schizophrenia patients aged 20–30 years, so as to avoid the confounding effects of age. Although the literature contains no data, only male patients were selected due to the possibility that gender might affect gene mRNA levels. Additional studies that compare changes in activation of the mitochondrial complex according to gender and age are warranted.

The first-episode schizophrenia patients in the present study were drug naïve, but the chronic schizophrenia patients had a history of antipsychotic drug use. Various effects of antipsychotics on mtETC have been reported [27-32]. Positive history of antipsychotic drug use might have effects on gene mRNA levels. To minimize this possibility, the present study’s chronic schizophrenia patients did not use any drug for 3 months prior to beginning the study.

## Conclusions

In conclusion, the present findings indicate that mRNA levels of complex I genes located in the mtETC might become a possible peripheral marker for the diagnosis of schizophrenia. Additionally, the findings show that there was a positive correlation between gene mRNA levels and psychotic symptomatology, especially positive symptoms. Long-term follow-up of schizophrenia patients to monitor changes in gene mRNA levels during different episodes of schizophrenia could help to clarify the etiopathogenesis of schizophrenia. Likewise, studies that compare changes in gene mRNA levels in schizophrenia patients that do and do not respond to drug treatment might further elucidate the disease’s etiology and the usefulness of activation of the mitochondrial complex as a biomarker for schizophrenia.
